# Dynamic construction of gut microbiota may influence allergic diseases of infants in Southwest China

**DOI:** 10.1186/s12866-019-1489-4

**Published:** 2019-06-10

**Authors:** Xi Shen, Maolin Wang, Xiao Zhang, Miao He, Ming Li, Guo Cheng, Chaomin Wan, Fang He

**Affiliations:** 1 Department of Nutrition, Food Hygiene and Toxicology, West China School of Public Health and Healthy Food Evaluation Research Center, Sichuan University, No.17 People’s South Road, Chengdu, Sichuan 610041 People’s Republic of China; 2 West China Second University Hospital, Sichuan University, 610041 Chengdu, Sichuan People’s Republic of China; 3 Department of Pediatrics of Western China Second Hospital of Sichuan University, Key Laboratory of Birth Defects and Related Diseases of Women and Children, 610041 Chengdu, Sichuan People’s Republic of China

**Keywords:** Allergic diseases, Dynamic process, Gastrointestinal microbiome, Infant, RNA,Ribosomal,16S, High-throughput nucleotide sequencing

## Abstract

**Background:**

Gut microbes have been suggested as the possible targets in the management of allergic diseases. However, the way in which these microbes influence allergic diseases remain unclear. Forty-seven full-term newborns were selected from a 1000-infant birth cohort. Among them were 23 allergic infants, whereas 24 infants were healthy without allergic symptoms at 1 year of age. Two hundred and sixty-four fecal samples were collected at 7 time points following their birth. These fecal samples were microbiologically analyzed using 16S rRNA gene sequencing. The dynamic processes involved in gut microbiota diversity and composition in the tested infants were constructed.

**Results:**

Healthy infants demonstrated more statistical differences in longitudinal changes in the alpha diversity of their microbiota at the time points compared with day 0 (meconium) than did allergic infants. Analysis of beta diversity showed that the fecal microbiota of days 0 and 2 comprised different communities in healthy infants, and that there were three separate communities in the fecal microbiota of day 0 of the healthy infants, those of day 2 of the healthy infants, and those of days 0–2 of the allergic infants. The relative abundance of dominant gut microbiota at phylum level varied at different time points in the healthy and diseased groups. *Bifidobacterium*, *Escherichia-Shigella*, *Streptococcus*, *Clostridium_sensu_stricto_1*, *Akkermansia* and *Erysipelatoclostridium* were significantly different between the healthy and diseased groups at a different time points.

**Conclusion:**

The dynamic construction processes of gut microbiota during early life might be associated with the occurrence of long-term allergic diseases, with the first month following birth potentially being the most critical.

**Electronic supplementary material:**

The online version of this article (10.1186/s12866-019-1489-4) contains supplementary material, which is available to authorized users.

## Background

In recent years, allergic diseases have been listed by the World Health Organization as one of the “three major diseases of the 21^st^ century” and listed by the World Allergy Organization (WAO) as “a public health issue of global concern”. In 2006, the WAO published an epidemiological survey of allergic diseases in 33 countries [1]. The results showed that approximately 22% of the 1.39 billion people in these countries had different types of allergic diseases and/or disorders [1]. Over the past 40 years, allergic diseases, such as asthma, allergic rhinitis, and food allergies, have become increasingly common in developed countries. Although these diseases rarely cause death, they do result in huge economic costs [2]. Currently, with the development of China’s social economy and living standards, the outlook for allergic diseases in China is not optimistic. Studies have demonstrated that the prevalence of asthma in urban children < 14 years old in China is 2.32% on average [3], whereas the incidence of infant eczema is about 64.8% [4]. Allergic diseases undergo a dynamic development process and could be lifelong. Infants and young children primarily suffer from food allergies and atopic dermatitis that can last for several years and may gradually develop into allergic rhinitis and asthma with age. Therefore, prevention is of particular importance [5].

Strachan have proposed an atopy-related “hygiene hypothesis” as early as in 1989 that suggested that with the living and hygiene standards improved and families becoming smaller, the reduced microbial exposure at the beginning of life increased the risk of allergic diseases in childhood [6, 7]. Subsequent research has revealed that “Thl/Th2 cell deviation” might be one of the immunological bases of the hygiene hypothesis [6]. An “Excessive hygiene” environment reduces the chances of the immune system of infants and young children being stimulated by antigens during early life, which might limit the development and maturation of their immune system, causing immune responses to shift to Th2, and subsequently increasing the secretion of the cytokines IL-4, IL-5 and IL-13. This further stimulates B cells to generate IgE, and activates eosinophil proliferation which releases inflammatory factors that further leads to allergic diseases [8–12].

The recently developed “microbiota hypothesis,” however, appears to be a more appropriate postulation [13]. Increasing evidence strongly suggests that several allergy-protective effects arise via altering the gut microbiota of children during the first year of life [14]. Several studies have shown that the use of medications that could directly cause intestinal dysbiosis, increased the risk for allergy development via altering the human microbiome [15–17]. Moreover under-representation of specific bacterial taxa has been revealed to be involved in the development of allergic diseases [18, 19]. However, the conclusions of various studies remain inconsistent, which possibly suggests variable microbial differences depending on the sampling time points employed [20].

The prenatal and early postnatal periods from conception to 2 years of age represent a critical window of early childhood growth and development during which the metabolic, endocrine, neural, and immune pathways are rapidly maturing. Trillions of microbes (microbiota) and their genes (microbiome) that reside within the human body colonize and stabilize during the first 2 years of life [21]. Evidence suggests that the microbial colonization in the human body during early life plays a critical role and that the disruption of this optimal microbial succession contributes to lifelong and intergenerational deficits in growth and development [22].

The objectives of this study were to evaluate the differences in the dynamic construction processes of gut microbiota during early life, to investigate whether these changes are associated with the occurrence of allergic diseases in childhood and throughout life, and determine whether there might be any key gut microbes associated with allergic diseases.

## Results

### Subjects

Participant characteristics were described in Table 1. The healthy group comprised 16 males and 8 females (mean birth weight, 3457 ± 393 g). The diseased group comprised 9 males and 14 females (mean birth weight, 3252 ± 346 g). No significant differences were observed between the baseline characteristics of the two groups.

**Table 1:**
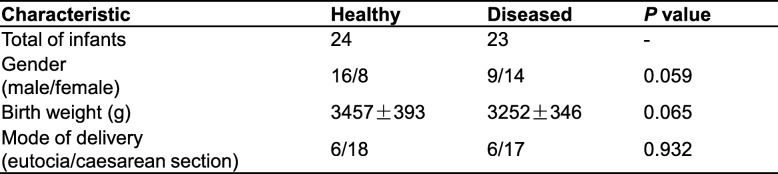
Characteristics of study population

Values are presented as number of people or mean ± SD, tested for differences between the groups using chi-square test. No significant differences were observed between two groups

### Factors influencing participants

No differences were observed between factors influencing the healthy and diseased groups, such as delivery mode, family allergy history, probiotic or prebiotic consumption during pregnancy by the mother, short-term antibiotic consumption during or after delivery by the mother, or antibiotic consumption within 1 month after birth by the infant (Additional file 1: Table S2). Furthermore, no differences were observed in terms of feeding factors, including colostrum consumption, breast milk feeding, milk powder feeding, complementary feeding and probiotic or prebiotic consumption by the infant (Additional file 1: Table S3).

### Reads and OTUs

Overall, 9,946,603 high-quality reads (679,300 reads eliminated during quality control) were obtained from the 264 samples, with 37,677 ± 8742 high-quality reads per sample, which were clustered into 3309 OTUs.

### Differences in variation over time of alpha diversity in healthy and diseased groups

**Fig. 1:**
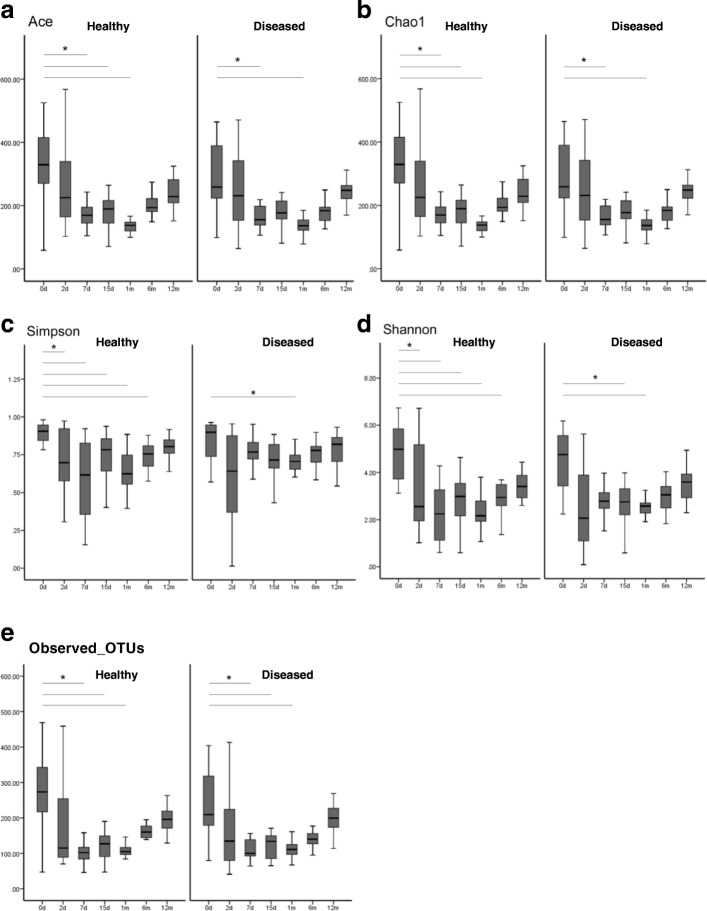
Differences in variation over time of alpha diversity between healthy and diseased groups using different measure indexes. Ace, Chao1 and Observed OTUs indexes reflect species richness, and Shannon or Simpson indexes reflect species diversity. **a**, **b**, **c**, **d**, **e** Kruskal-wallis test was used for analysing differences among time groups in the healthy and diseased groups, respectively. Values are presented as mean ± S.E.M. * *P* < 0.05 compared to day 0 samples

Five alpha diversity calculated indexes, which included Ace, Chao1, Shannon, Simpson and Observed OTUs, reflect richness and diversity of the gut microbiota. Alpha diversity indexes varied over time in both healthy and diseased groups and showed a similar trend. In day 0 samples (meconium), richness and diversity were at a higher level but showed a downward trend from day 2 to day 7 after birth. Subsequently, richness and diversity showed a slight increase by day 15, following which a declining trend was noted at 1 month after birth. Finally, richness and diversity continued to increase until 12 months following birth; however, it did not return to the level of the day 0 samples (meconium) (Fig. 1).

In the longitudinal variation of the Ace, Chao1, Shannon, and Simpson indexes, on comparing every time point with day 0, statistical differences were observed at more time points in the healthy group than the diseased group (Fig. 1). The observed OTUs showed a similar statistical difference in the longitudinal variation between the two groups. No statistical differences were observed in the five indices between the two groups at any time point (Additional file 1: Figures S1, S2, S3, S4, S5, S6 and S7).

### Differences in variation over time of beta diversity in healthy and diseased groups

Partial least squares discriminant analysis (PLS-DA) was performed based on time points and diseased groups; the results reflected beta diversity, which represent the differences in community structure between groups. PLS-DA can effectively distinguish observations between groups and detect the variables that influence the differences between groups. The coordinate axes represent the two components (PC1 and PC2) that can appropriately reflect the sample differences. In the PC1 dimension, the inter-group samples can be easily distinguished, whereas in the PC2 dimension, parts of the samples within the group can be differentiated.

**Fig. 2:**
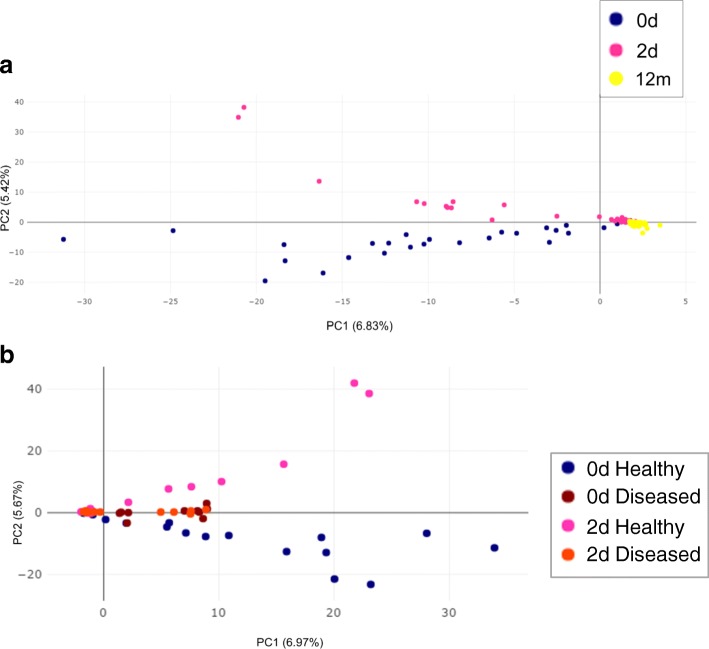
Beta diversity of day 0, day2 and month 12. PLS-DA was carried out according to time group, and the results reflect beta diversity which present differences in community structure between time groups. **a** Samples of day 0 (meconium), day 2 and month 12 were divided into three different communities. **b** Day 0 samples (meconium) of the healthy group, day 2 samples of the healthy group and day 0 to day 2 samples of the diseased group were divided into three different communities

Considering only time points, on dividing samples from day 0 (meconium) and day 2 into different communities, the PC2 contribution rate was 5.42% (Fig. 2a); furthermore, the samples of day 0 (meconium), day 2, and month 12 belonged to different communities, and the PC1 contribution rate was 6.83% (Fig. 2a). Samples from day 7 to month 12 could not be clearly divided using PLS-DA (Additional file 1: Figure S8a), but the community structure of each time point gradually became closer to resembling that of the 12-month samples (Additional file 1: Figure S8b).

Considering both time points and diseased groups, day 0 samples (meconium) of the healthy group, day 2 samples of the healthy group, and days 0–2 samples of the diseased group, were divided into three different communities, whereas day 0 samples (meconium) of the diseased group were difficult to distinguish from day 2 samples of the diseased group (Fig. 2b). Furthermore, day 7–month 12 samples of the healthy and diseased groups could not be distinguished (Additional file 1: Figure S9).

### Composition of fecal microbiota at the phylum level varied over time in healthy and diseased groups

**Fig. 3:**
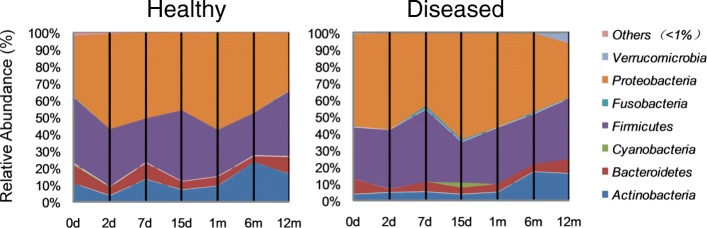
Composition of gut microbiota at the phylum level varied over time in healthy and diseased groups. Phyla with the relative abundance of more than 1% are presented, and the lower abundant phyla are grouped as ‘Others

At the phylum level, the taxonomy-based analysis revealed that the fecal microbiota in infancy (0–1-year-olds) primarily comprised species belonging to the phyla *Firmicutes* and *Proteobacteria*, followed by those belonging to *Actinobacteria* and *Bacteroidetes.* The relative abundance of each phylum in month 1 following birth showed a variation in increases and decreases; the healthy group showed different trends in variation over time compared with the diseased group. However, after month 1, the trend of relative abundance in each group varied over time in a similar manner (Fig. 3).

**Fig. 4:**
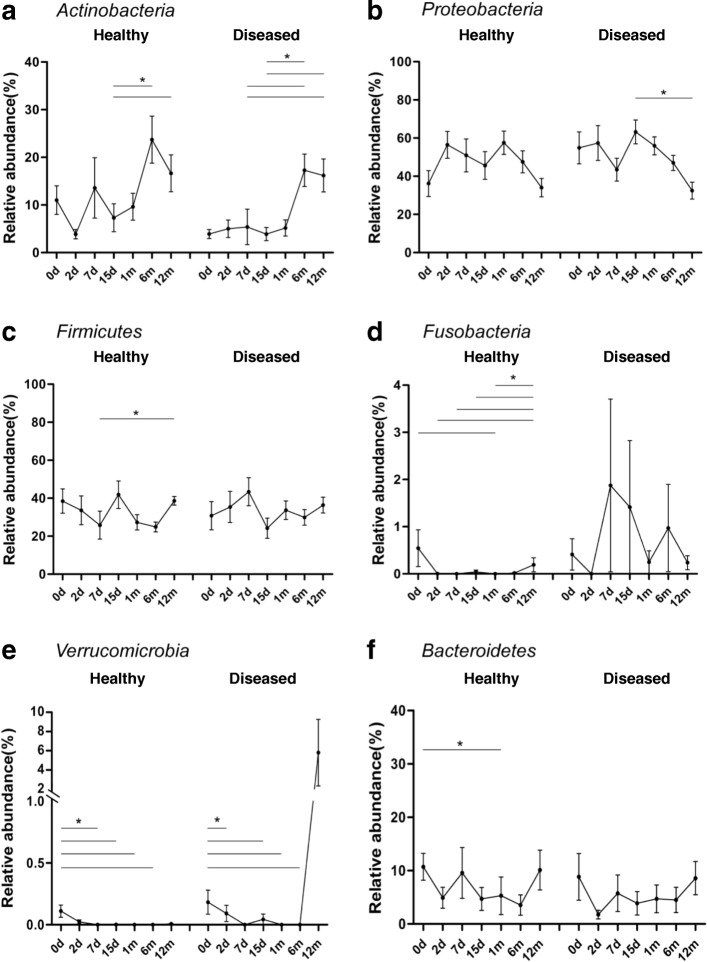
**a**, **b**, **c**, **d**, **e**, **f** Dominant microbiota at the phylum level varied over time in healthy and diseased groups. Kruskal-wallis test was used for analysing differences among time groups in the healthy and diseased groups, respectively. Values are presented as mean ± S.E.M. * *P* < 0.05 compared to samples of the time point

Figure 4 shows the changes in the dominant gut microbiota over time (Fig. 4). In the healthy group, a certain level of *Actinobacteria* colonization was observed in day 0 samples (meconium), with a relative abundance of 11.00%; the relative abundance decreased to 3.85% in day 2 samples, increased to 13.59% in day 7 samples, and decreased to 7.31% in day 15 samples, following which it continued to increase to 9.62% in month 1 samples, to 23.73% in month 6 samples, and finally decreased to 16.65% in month 12 samples. However, in the diseased group, the relative abundance of *Actinobacteria* in day 0 samples (meconium) was lower (3.90%) than the healthy group, but it was close to the level observed in day 2 samples of the healthy group. It showed slight fluctuation in the range of 3–5% and began to increase after 1 month, reaching a peak value of 17.28% in month 6 samples, and slightly decreasing to 16.19% in month 12 samples, which was close to the level observed in month 12 samples of the healthy group (Fig. 4a).

The relative abundance of another dominant bacterial phylum, *Proteobacteria*, varied with time and showed a different trend between the two groups. In the healthy group, the relative abundance of *Proteobacteria* was 36.18% in day 0 samples (meconium); it increased to 56.46% in day 2 samples, decreased to 45.60% in day 15 samples, and increased to 57.55% in month 1 samples, following which it continued to decrease to 34.00% in month 12 samples, which was similar to the level at day 0. The relative abundance of *Proteobacteria* in day 0 samples (meconium) of the diseased group was 54.89%, which was close to levels observed in the day 2 samples of the healthy group. This level of relative abundance was maintained until day 2 (57.41%), following which it slightly decreased; it was 43.47% in day 7 samples, gradually increasing to 63.21% in day 15 samples, and finally decreasing to 32.46% in month 12 samples, which was close to the level observed of day 0 samples (meconium) of the healthy group (Fig. 4b).

The variation of *Firmicutes* over time in the healthy group was different compared with the diseased group. In the healthy group, the relative abundance of *Firmicutes* showed a decreasing trend following birth—from 38.48% in day 0 samples (meconium) to 25.83% in day 7 samples. Subsequently, the relative abundance began to increase to 41.84% in day 15 samples, following which it continued to decrease to 24.89% in month 6 samples. Finally, before month 12, it increased to 38.60%, which was similar to the level observed in day 0 samples (meconium). In the diseased group, the relative abundance of *Firmicutes* demonstrated an increasing trend after birth from 30.79% in day 0 samples (meconium) to 43.41% in day 7 samples, following which it showed a downward trend until day 15 and reached a trough (24.23%); it gradually increased to 36.36% in month 12 samples, which was similar to the levels observed in day 0 samples (meconium) of the healthy group (Fig. 4c).

*Fusobacteria* were present at a certain level in day 0 samples (meconium) of the two groups (healthy, 0.55%; diseased, 0.41%). In samples from day 2 to month 6 of the healthy group, the relative abundance of *Fusobacteria* was very low, with a small amount present in month 12 samples (0.19%). In the diseased group, the relative abundance of *Fusobacteria* extensively fluctuated in samples from day 2 to month 12; however, in month 12 samples, it returned to levels similar to that observed in month 12 samples of the healthy group (0.24%) (Fig. 4d).

**Fig. 5:**
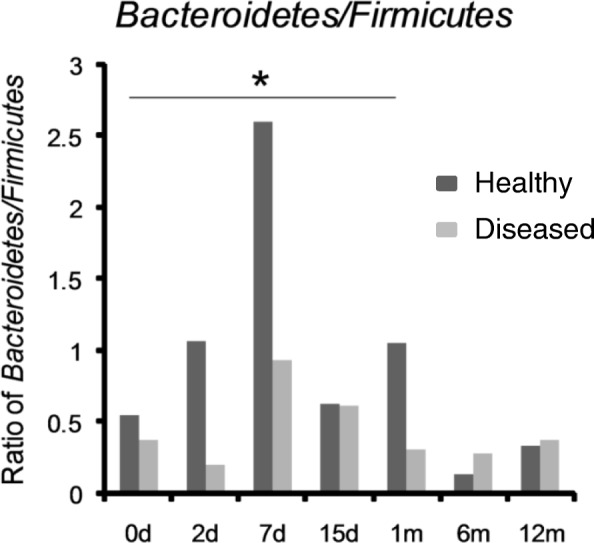
*Bacteroidetes*/*Firmicutes* ratio. *Bacteroidetes/Firmicutes* ratio of month 1 samples was significantly higher than that of day 0 samples (meconium) in healthy group. Month 1 samples in healthy group, 1.06; day 0 samples (meconium) in healthy group, 0.55. * *P* < 0.05 compared to samples of day 0 in healthy group

Furthermore, in the healthy group, a small amount of *Verrucomicrobia* colonization (0.11%) was found in day 0 samples (meconium), whereas almost none was found after day 2. In the diseased group, a small amount of *Verrucomicrobia* colonization (0.18%) was found in day 0 samples (meconium), which gradually decreased and reappeared in a relatively large proportion (5.8%) in month 12 samples (Fig. 4e). *Bacteroidetes* varied in a similar manner over time in both groups (Fig. 4f), whereas in the healthy group, the ratio of *Bacteroidetes/Firmicutes* in month 1 samples was significantly higher than that in day 0 samples (meconium) (1.06 vs 0.55, *P*<0.05) (Fig. 5).

### Comparison of gut microbiota in healthy and diseased groups

In the day 0 (meconium) samples, the relative abundance of taxa belonging to the phylum *Actinobacteria* (*P* = 0.024), *Cyanobacteria* (*P* = 0.030), and *Saccharibacteria* (*P* = 0.003) in the healthy group was significantly higher than that in the diseased group. In day 7 samples, the relative abundance of the phylum *Fusobacteria* (*P* = 0.046) in the healthy group was significantly lower than that in the diseased group. At day 15, a significant difference in unclassified OTUs was observed between both groups, whereas at month 1, no significant difference was observed between both groups. In month 6 samples, the relative abundance of the phylum *Fusobacteria* (*P* = 0.000) in the healthy group was significantly lower than that in the diseased group, whereas in month 12 samples, the relative abundance of the phylum *Verrucomicrobia* (*P* = 0.015) in the healthy group was significantly lower than that in the diseased group (Table 2).

**Table 2:**
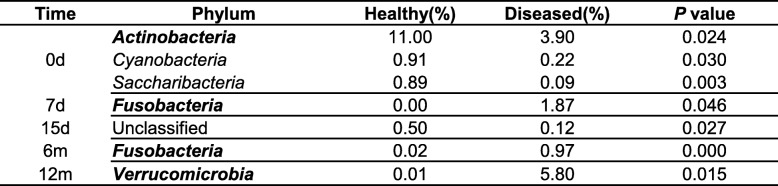
Comparison of gut microbiota at phylum level between healthy and diseased groups

Values are presented as percentage of relative abundance, tested for differences between the groups using Wilcoxon rank-sum test. Only phyla presenting significant differences are listed in the table. A *P*-value < 0.05 was defined as statistically significant

At the genus level, significant differences in relative abundance between the two groups at the same time point are listed in Table 3. In particular, the relative abundance of taxa belonging to the genus *Bifidobacterium* (*P* = 0.047) in day 0 samples (meconium) of the healthy group was significantly higher than that of the diseased group; however, the relative abundance of the genus *Escherichia–Shigella* (*P* = 0.038) in day 0 samples (meconium) of the healthy group was significantly lower than that in the diseased group. In day 7 samples, the relative abundance of the genus *Streptococcus* (*P* = 0.048) in the healthy group was significantly lower than that in the diseased group. In day 15 samples, the relative abundance of the genus *Clostridium_sensu_stricto_1* (*P* = 0.033) in the healthy group was significantly higher than that in the diseased group. In month 12 samples, the relative abundance of the genus *Akkermansia* (*P* = 0.012) in the healthy group was significantly lower than that in the diseased group, and the relative abundance of the genus *Erysipelatoclostridium* (*P* = 0.038) in the healthy group was significantly higher than that in the diseased group (Table 3).

**Table 3:**
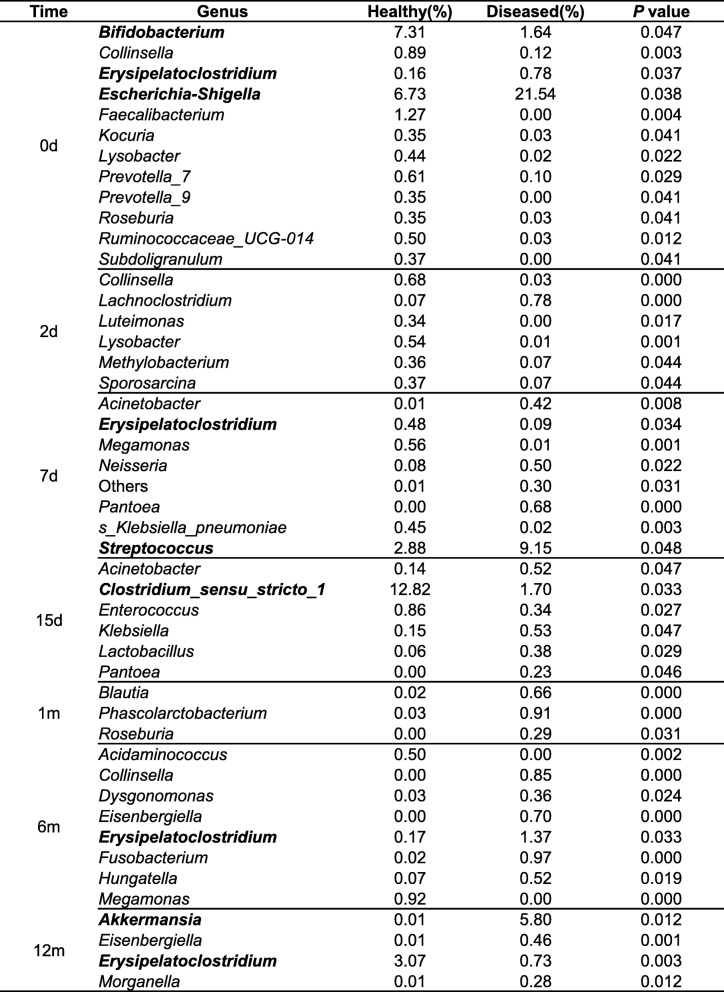
Comparison of gut microbiota at genus level between healthy and diseased groups

Genera with the relative abundance are presented as mean and compared between the healthy and diseased groups using Wilcoxon rank-sum test. The lower abundant genera are grouped as ‘Others’. Only genera presenting significant differences are listed in the table. A *P*-value < 0.05 was defined as statistically significant

## Discussion

A increasing number of studies have found that gut microbiota during early life is associated with allergic diseases in childhood and even throughout life. The time period of these studies tended to concentrate on the 2 years following birth; however, because of the different sampling time points used in each study, the differences in gut microbiota found between the healthy and the allergic diseases groups were inconsistent [20]. Considering these previous studies, more scientific attention should be focused on the dynamic construction processes of gut microbiota during early life as a whole, instead of analyzing and comparing the difference between single species and/or communities of gut microbiota at single and independent time points. This would enable us to explore the mechanisms underlying the influence of gut microbes on allergic diseases in childhood and throughout life.

Several indexes were used to profile the structure of the fecal microbiota of the tested infants from different aspects. Ace, Chao1 and Observed OTUs could reflect species richness, and Shannon and Simpson indices might reflect species diversity. In the present study, these tested indices were higher in day 0 samples (meconium), and gradually declined over month 1, except for fluctuations observed on day 7 and 15. Subsequently, these indices increased from month 1 to month 12 in both healthy and diseased groups. However, trends in alpha diversity variations during early life were consistent between healthy and diseased groups within 1 year of birth. Because the meconium is more likely to reflect the gut microbiota of the fetus in the uterus, we inferred that the high richness and diversity observed in the meconium samples may have been derived from the mother during the prenatal period, at least partly. On the other hand, this also may be attributed to the special environment immediately following birth. Alpha diversity indices initially decreased to a lower level after day 0, before increasing again, which we defined here as a process of “reconstruction”. No alpha diversity differences were observed between healthy and diseased groups within 1 year of age at any time point in the present study. Nevertheless, more statistical differences were observed in the dynamic processes of alpha diversity variations in the healthy group, indicating that the degree of alpha diversity fluctuation is greater in the healthy group than the diseased group within 1 year of age. Therefore, these results indicate that the infants in the healthy group, at least in the present study have been exposed to additional microbial stimulation during their characteristic dynamic processes, and the intestinal microbiota of their mother in the prenatal period might be also involved. These findings were partly supported by our recent studies using an animal model, where mice offspring exposed to antibiotics during the prenatal period demonstrated stronger inflammatory responses despite being delivered by Cesarean section (submitted for publication). On the other hand, the environmental factors following birth may also be associated with the immune mechanism; however, this requires further research. Further studies should be conducted focusing on the mechanism underlying the influence of the maternal gut microbiota during the prenatal period on the health of the offspring.

Currently, there are insufficient studies on the dynamic processes of alpha diversity variations in the gut microbiota during early life. The present study brings additional evidences that can assist further studies in this field. Existing studies, using 16S rRNA sequencing or gel-based techniques, have found that lower alpha diversity indices are associated with allergic diseases in infants less than 1 year of age [23]. It is assumed that microbiota diversity contributes to the development of regulatory T cells, cytokines, and complement networks to assist in the prevention of autoimmune conditions, allergies, and eczema [7]. It is possible that dysbiosis and lower gut microbiota diversity in infancy could be associated with failures in allergen tolerance [23]. The large fluctuations of gut microbiota alpha diversity observed in healthy infants may be more effective in stimulating the immune system, thereby promoting allergen tolerance and further reduction of the risk of allergic diseases, although the immune mechanism underlying this requires further research.

In the present study, the beta diversity analysis indicated that the period from day 0 to day 2 is a more accurate critical window for the dynamic construction processes of gut microbiota during early life. It showed that the dynamic construction processes of the infant gut microbiota within 1 year after birth were divided into two stages: days 0–2 and day 7–month 12. Importantly, in the stage of days 0–2, the dynamic processes of the gut microbiota in healthy and diseased groups were different. In infants of the healthy group, there was a large transition of the gut microbiota structure from the day 0 (meconium) to day 2, which might be related to the process of reconstruction, as defined in the present study.

In terms of the dynamic construction processes of specific gut microbiota at the phylum level, certain microbes found in healthy infants experienced the process of reconstruction too. The abundance of *Actinobacteria* in the meconium of infants in the healthy group was significantly higher than that of meconium from those in the diseased group. Higher *Actinobacteria* in the meconium of the healthy group could not be maintained and immediately decreased at day 2, with large dynamic fluctuations within 1 month. The abundance of *Actinobacteria* in the meconium of the diseased group started at a lower level, which was similar to that in day 2 samples of the healthy group, without the process of reconstruction. Dynamic fluctuations in the diseased group within 1 month were less than that in the healthy group. The abundance of *Actinobacteria* in both healthy and diseased groups was consistently close to a stable situation at month 12. These results indicate that infants in the healthy group may have been exposed to more *Actinobacteria,* which might have been derived from their mother, whereas infants in the diseased group might not have received this maternal transmission of *Actinobacteria*. Healthy infants experience a characteristic process of reconstruction within 1 month after birth, reaching a mature situation at month 12. Infants with allergy lack this reconstruction process; although there is a construction process in these infants. The reconstruction process might cause more fluctuations in the gut microbiota within 1 month after birth, which could be more conducive to avoiding allergic diseases. The dynamic construction processes of *Proteobacteria* and *Firmicutes* exhibited a similar tendency to *Actinobacteria*, showing the characteristic process of reconstruction and fluctuations within 1 month after birth in healthy infants.

However, the greater fluctuations in *Fusobacteria* and *Verrucomicrobia* may be detrimental to the prevention of allergic diseases. The abundance of *Fusobacteria* and *Verrucomicrobia* in the meconium of healthy infants was similar to that found in the meconium of infants with allergy. These two phyla were subsequently reduced in healthy infants with fewer fluctuations within 1 month after birth, whereas there were considerable fluctuations in infants with allergy. The abundance of *Fusobacteria* was relatively higher in infants with allergy than healthy infants at day 7 and at month 6, whereas the abundance of *Verrucomicrobia* was relatively higher in infants with allergy at month 12. The reason for these phenomena might be associated with the interactions of different bacteria and the different mechanisms of immune system stimulation, which require further research. The construction process of the relatively abundant phylum of *Bacteroidetes* was similar in both healthy infants and infants with allergy, whereas the ratio of *Bacteroidetes: Firmicutes* in month 1 samples of infants with allergy was higher than that of healthy infants.

The present study found certain differences in microbes between the healthy and diseased groups at the genus level. Several studies have found that fewer *Bifidobacterium* were present in infants with allergy aged less than 1 year than that in healthy infants; conversely, *Clostridium* was higher in infants with allergy than in healthy infants [24–27]. The results of the present study were consistent with the fact that *Bifidobacterium* levels were higher in the meconium of healthy infants than that of infants with allergy; however, the difference was that the *Clostridium_sensu_stricto* levels were higher in healthy infants than in infants with allergy at day 7. These results are similar to a Swedish study on food allergies and allergic asthma [28]. This may be attributed to different early life factors, such as feeding mode or antibiotic use.

Previous studies have found that the colonization of *Streptococcus pneumoniae* in 1-month-old infants’ hypopharyngeal region was associated with persistent wheeze, acute exacerbation of wheeze, and hospitalization for wheeze by age 5; asymptomatic colonization with *Streptococcus* was believed to be a strong predictor of asthma [29, 30]. In the present study, there was significantly less *Streptococcus* in the gut of healthy infants compared with infants with allergy at day 7, which suggests that these microbes might be correlated with a risk of allergic diseases.

Recently, the *Akkermansia* genus has received much attention because these organisms have been found to be greatly involved in human health and various diseases. For example, a decrease in *Akkermansia muciniphila* in the human intestine was associated with an increased risk of type 2 diabetes and atopic dermatitis [28, 31]. However, the present study observed that *Akkermansia* levels were higher in infants with allergy than healthy infants at month 12. Further research should focus on the role of these intestinal microbes in the development of allergies in infants.

Additionally, we found that a common pathogen taxa, *Escherichia–Shigella*, was present in higher abundance in the meconium of infants with allergy than that of healthy infants. *Escherichia–Shigella* is one of the top four pathogens that cause moderate-to-severe diarrhea in African and South Asian children [32], and these intestinal microbes might be considered as possible targets in the management of allergic disease in infants.

*Erysipelatoclostridium* was present in significantly different amounts in day 0 (meconium), day 7, month 6, and month 12 samples in healthy infants compared with allergic infants. Dairy intake was positively associated with *Erysipelatoclostridium* and has been linked to metabolic syndrome in humans, and the up regulation of small intestinal glucose and fat transporters, which results in enhanced diet-induced obesity, in animal studies [33]. Further studies should be conducted to analyze possible associations between microbes and allergic diseases in infants.

The results of this study indicate that although specific microbiota might be associated with allergic diseases, individual bacteria showed different results compared with existing studies—this might have been affected by ethnic group, geographical region, and lifestyle habits. Further research is needed. *Bifidobacterium*, *Clostridium_sensu_stricto*, *Streptococcus*, *Escherichia–Shigella*, *Erysipelatoclostridium*, and *Akkermansia* have a probable predictive effect on the risk of allergic diseases in children in southwestern China during their first year of life. However, the health prediction effects of a single specific microbiota may be very weak; additional focus on the dynamic construction processes of gut microbiota and the structure of gut microbiota during early life is warranted.

## Conclusions

The dynamic construction processes of gut microbiota during early life may be associated with the occurrence of long-term allergic diseases. The period from day 0 to month 1 after birth may be a more accurate critical window for the prevention of allergic diseases. The characteristic reconstruction of *Actinobacteria*, *Proteobacteria*, and *Firmicutes,* or fluctuations of *Fusobacteria* and *Verrucomicrobia* in the first 2 days after birth, may be associated with the occurrence of allergic diseases. Certain intestinal microbes, including *Bifidobacterium*, *Clostridium_sensu_stricto*, *Streptococcus*, *Escherichia–Shigella*, *Erysipelatoclostridium,* and *Akkermansia* may play an important role in allergic diseases of infants, at least those born in southwestern China. However, further studies focusing on the underlying mechanisms behind these findings as well as the impact of environmental factors, such as delivery mode and feeding mode, on the gut microbiota and allergic diseases during early life are warranted.

## Methods

### Study design, subjects and sample collection

This is a nested case-control study. Overall, 47 full-term newborns were selected from a 1000-infant birth cohort from the West China Second University Hospital (West China Women’s and Children’s Hospital), according to the number of fecal samples collected and occurrence of allergic disease. The diseased group comprised 23 infants who were diagnosed in the hospital with at least one allergic disease and/or disorder, including allergic eczema, food allergies, drug allergies, and allergic asthma within 1 year of age, based on National Clinical Guidance specifically for infants’ allergic disease prevention, diagnosis, and treatment [34]. The remaining 24 infants who had not been diagnosed with allergic disease within 1 year of age were the healthy group. Infants with serious or congenital diseases or those who had undergone invasive intestinal examination or treatment were excluded. All participants’ guardians provided written informed consent to participate in the study.

All participants’ mothers completed questionnaires prior to delivery, 1 month after delivery, 6 months after delivery, and 1 year after delivery. The questionnaire included questions regarding family allergy history, probiotic or prebiotic consumption, infant feeding, and antibiotic use. Other basic information was obtained from medical records.

Fecal samples were collected from each infant at day 0 (meconium, 0–24 h), day 2 (24–48 h), day 7, and day 15 as well as at 1, 6, and 12 months following birth. Overall, 264 fecal samples were collected (Additional file 1: Table S1). The rate of collection of fecal samples per infant was > 80%. All fecal samples were stored at − 80 °C until DNA extraction was performed.

All procedures in the study were conducted in accordance with the Declaration of Helsinki and were reviewed and approved by the Ethics Committee of West China School of Public Health (No. 4 West China Teaching Hospital) of Sichuan University.

### DNA extraction and 16S rRNA sequencing

Fecal bacterial total DNA was extracted from fecal samples using a commercial DNA isolation kit (TIANamp Stool DNA Kit, TIANGEN, Beijing, China) according to the manufacturer’s protocol. DNA concentration and purity were analyzed using a Nanodrop 2000 ultraviolet-visible microspectrophotometer (Thermo-Fisher scientific, Inc., USA).

The 5′ ends of the primers were tagged with specific barcodes for each sample and sequencing universal primers. PCR amplification of the V3-V4 rRNA gene was performed using forward primer 338F 5′-ACTCCTACGGGAGGCAGCAG-3′ and reverse primer 806R 5′-GGACTACHVGGGTWTCTAAT-3′ (The amplicon size was 468 bp) [35]. The PCR products were confirmed using 2% agarose gel electrophoresis, purified using AMPure XT beads (Beckman Coulter Genomics, Danvers, MA, USA), and quantified using Qubit (Invitrogen, USA). The amplicon pools were prepared for sequencing, and the size and quantity of the amplicon library were assessed using an Agilent 2100 Bioanalyzer (Agilent, USA) and the Library Quantification Kit for Illumina (Kapa Biosciences, Woburn, MA, USA), respectively. PhiX Control Library (v3) (Illumina Inc., San Diego, CA) was combined with the amplicon library (expected at 30%). The libraries were sequenced using the 300-bp paired-end protocol with the standard Illumina sequencing primers on an Illumina MiSeq Instrument (Illumina Inc., San Diego, CA).

### Data preprocessing and sequence analysis

Demultiplexed FASTQ files were generated from base-calls using the Illumina bcl2fastq software (v1.8.42013). Low-quality reads were filtered using Trimmomatic (v0.36, 2016) [36], with a maximum of three mismatches per primer being allowed in the raw data. Paired-end reads were merged using FLASH (v1.2.11, 2014) [37]. The sliding window method was used to filter the double-ended fastq sequence to obtain a valid sequence with a minimum length restriction of 150 bases. Thereafter, the valid sequences were dereplicated and clustered into zero-radius operational taxonomic units (OTUs) using the USEARCH (v10.0.240_i86linux32, 2016) [38] that detected and removed chimeric sequences. Taxonomic assignments to the OTUs were performed using the USEARCH (v10.0.240_i86linux32, 2016) based on the Silva Living Tree Project v123 database (v123, 2015) [39]. Taxonomic assignments were considered reliable when bootstrap confidence values were > 0.75. Subsequently, an OTU abundance table and a phylogenetic tree were generated. Alpha diversity (Ace, Chao1, Shannon, Simpson, Observed OTUs) was calculated using QIIME (v1.9.1, 2015) [40]. And beta diversity (Bray Curtis, weighted and unweighted UniFrac) was estimated using the Phyloseq package (v1.20.0, 2017) [41] and visualized using the R program (v3.4.1, 2017).

### Statistical analysis

SPSS 22.0 was used for statistical analysis. Data are presented as mean ± standard deviation or mean ± S.E.M. The differences between basic information and factors influencing the healthy and diseased groups were analyzed using chi-square test. Mann–Whitney U-test was used for assessing differences between two groups at one time point. Kruskal–Wallis test was used for analyzing differences among time periods in the healthy and diseased groups. Differences with *P* < 0.05 were considered significant. Beta diversity was clustered using the partial least squares discriminant analysis (PLS-DA) and visualized using the R program (v3.4.1).

## Additional file

Additional file 1: **Table S1.** Number of faecal samples collected from the 47 infants in different time points of the study. **Table S2.** Comparison of influences in early life between healthy and diseased groups. **Table S3.** Comparison of feeding factors between healthyand diseasedgroups. **Figure S1.** Comparison of alpha diversity indexes at day 0 in the healthy and diseased groups. **Figure S2.** Comparison of alpha diversity indexes at day 2 in the healthy and diseased groups. **Figure S3.** Comparison of alpha diversity indexes at day 7 in the healthy and diseased groups. **Figure S4.** Comparison of alpha diversity indexes at day 15 in the healthy and diseased groups. **Figure S5.** Comparison of alpha diversity indexes at month 1 in the healthy and diseased groups. **Figure S6.** Comparison of alpha diversity indexes at month 6 in the healthy and diseased groups. **Figure S7.** Comparison of alpha diversity indexes at month 12 in the healthy and diseased groups. **Figure S8.** Beta diversity of day 7 to month 12. PLS-DA was carried out according to time group. a Samples from day 7 to month 12 could not be clearly divided by PLS-DA. b Trend of beta diversity variation from day 7 to month 12. **Figure S9.** Beta diversity of the healthy and diseased groups. PLS-DA was carried out according to time and diseased groups, and the results reflect beta diversity which is about differences in community structure between these groups. In day 7 to month 12, samples of the healthy and diseased groups could not be distinguished. (PDF 761 kb)

## Abbreviation

OTU: Operational taxonomic unit
