# Supplementary material for: Dynamic construction of gut microbiota may influence allergic diseases of infants in Southwest China
Source: BMC Microbiol. 2019 Jun 10;19:123. doi: 10.1186/s12866-019-1489-4 (PMC6558729; doi:10.1186/s12866-019-1489-4)
Supplement: Supplementary file 1 — Table S1. Number of faecal samples collected from the 47 infants in different time points of the study. Table S2. Comparison of influences in early life between healthy and diseased groups. Table S3. Comparison of feeding factors between healthyand diseasedgroups. Figure S1. Comparison of alpha diversity indexes at day 0 in the healthy and diseased groups. Figure S2. Comparison of alpha diversity indexes at day 2 in the healthy and diseased groups. Figure S3. Comparison of alpha diversity indexes at day 7 in the healthy and diseased groups. Figure S4. Comparison of alpha diversity indexes at day 15 in the healthy and diseased groups. Figure S5. Comparison of alpha diversity indexes at month 1 in the healthy and diseased groups. Figure S6. Comparison of alpha diversity indexes at month 6 in the healthy and diseased groups. Figure S7. Comparison of alpha diversity indexes at month 12 in the healthy and diseased groups. Figure S8. Beta diversity of day 7 to month 12. PLS-DA was carried out according to time group. a Samples from day 7 to month 12 could not be clearly divided by PLS-DA. b Trend of beta diversity variation from day 7 to month 12. Figure S9. Beta diversity of the healthy and diseased groups. PLS-DA was carried out according to time and diseased groups, and the results reflect beta diversity which is about differences in community structure between these groups. In day 7 to month 12, samples of the healthy and diseased groups could not be distinguished. (PDF 761 kb) [file 12866_2019_1489_MOESM1_ESM.pdf]

Supplementary data

Table S1

Number of faecal samples collected from the 47 infants in different time points of the study

**Group (n)**	**Faecal samples**						
	0d	2d	7d	15d	1m	6m	12m
Total (n=47)	28	39	37	41	41	36	42
Healthy (n=24)	16	21	18	22	20	15	22
Diseased (n=23)	12	18	19	19	21	21	20

Values are presented as number of people.

Table S2

Comparison of influences in early life between healthy and diseased groups

**Event**	**Healthy(%)**	**Diseased(%)**	***P* value**
Caesarean section	75	73.9	0.932
Mother history of allergies	41.7	34.8	0.627
Family history of allergies (except mother)	54.2	56.5	0.871
Mother taking probiotics or prebiotics during pregnancy	91.7	73.9	0.137
Mother using short-term antibiotics during or after delivery	75	82.6	0.724
Infant using antibiotics within one month after birth	12.5	13	0.999

Values are presented as percentage of subjects, tested for differences between the groups using chi-square test. A *P*-value <0.05 was defined as statistically significant. No significant differences were observed between two groups.

Table S3

Comparison of feeding factors between healthy and diseased groups

Feeding		Healthy(%)	Diseased(%)	*P* value
Colostrum		83.3	82.6	1.000
Breast milk	0d	25	25	1.000
	2d	61.9	83.3	0.138
	7d	83.3	100	0.105
	15d	86.4	94.7	0.61
	1m	85	95.2	0.343
	6m	60	76.2	0.465
	12m	36.4	25	0.426
Formula	0d	6.3	16.7	0.56
	2d	66.7	61.1	0.718
	7d	77.8	63.2	0.331
	15d	77.3	68.4	0.524
	1m	80	57.1	0.116
	6m	60	47.6	0.463
	12m	72.7	90	0.243
Complementary feeding	6m	93.3	90.5	1.000
	12m	100	100	-
Probiotics and prebiotics	0d-1m	45.8	60.9	0.302
	6m	66.7	76.2	0.709
	12m	63.6	75	0.426

Values are presented as percentage of subjects, tested for differences between the groups using chi-square test. A *P*-value <0.05 was defined as statistically significant. No significant differences were observed between two groups.

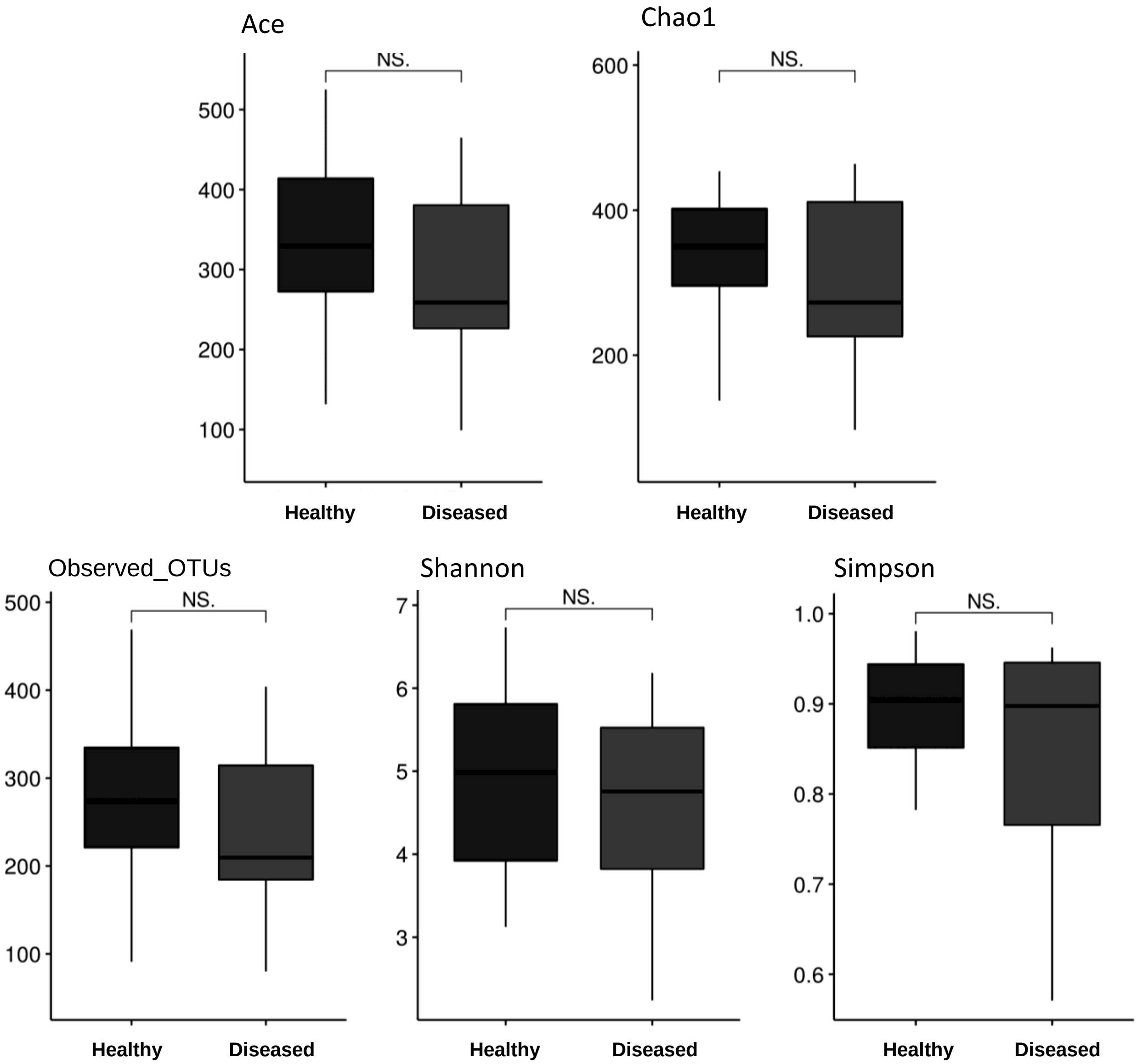

Figure S1

Comparison of alpha diversity indexes at day 0 in the healthy and diseased groups. No significant differences of were observed between two groups.

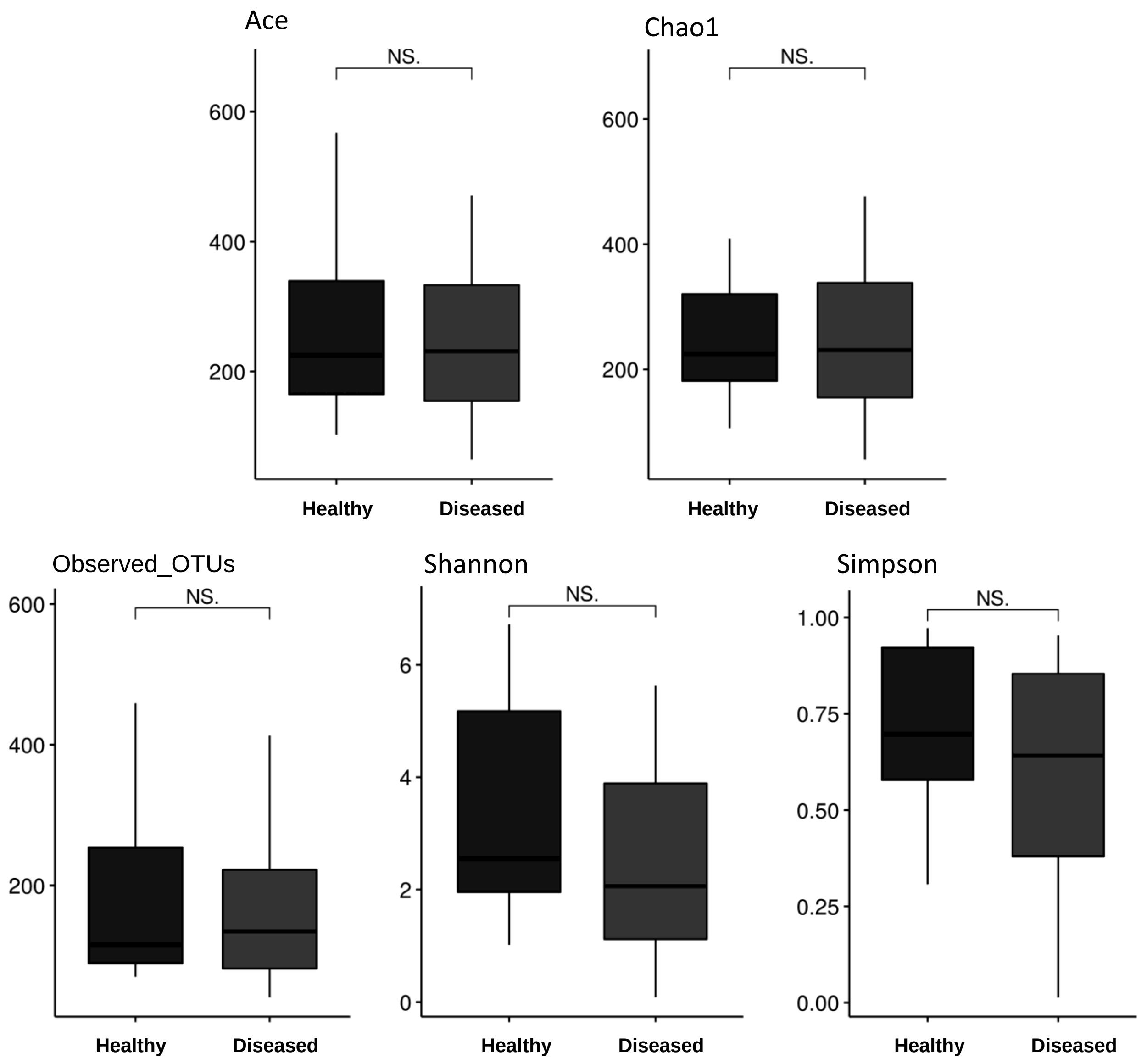

Figure S2

Comparison of alpha diversity indexes at day 2 in the healthy and diseased groups. No significant differences of were observed between two groups.

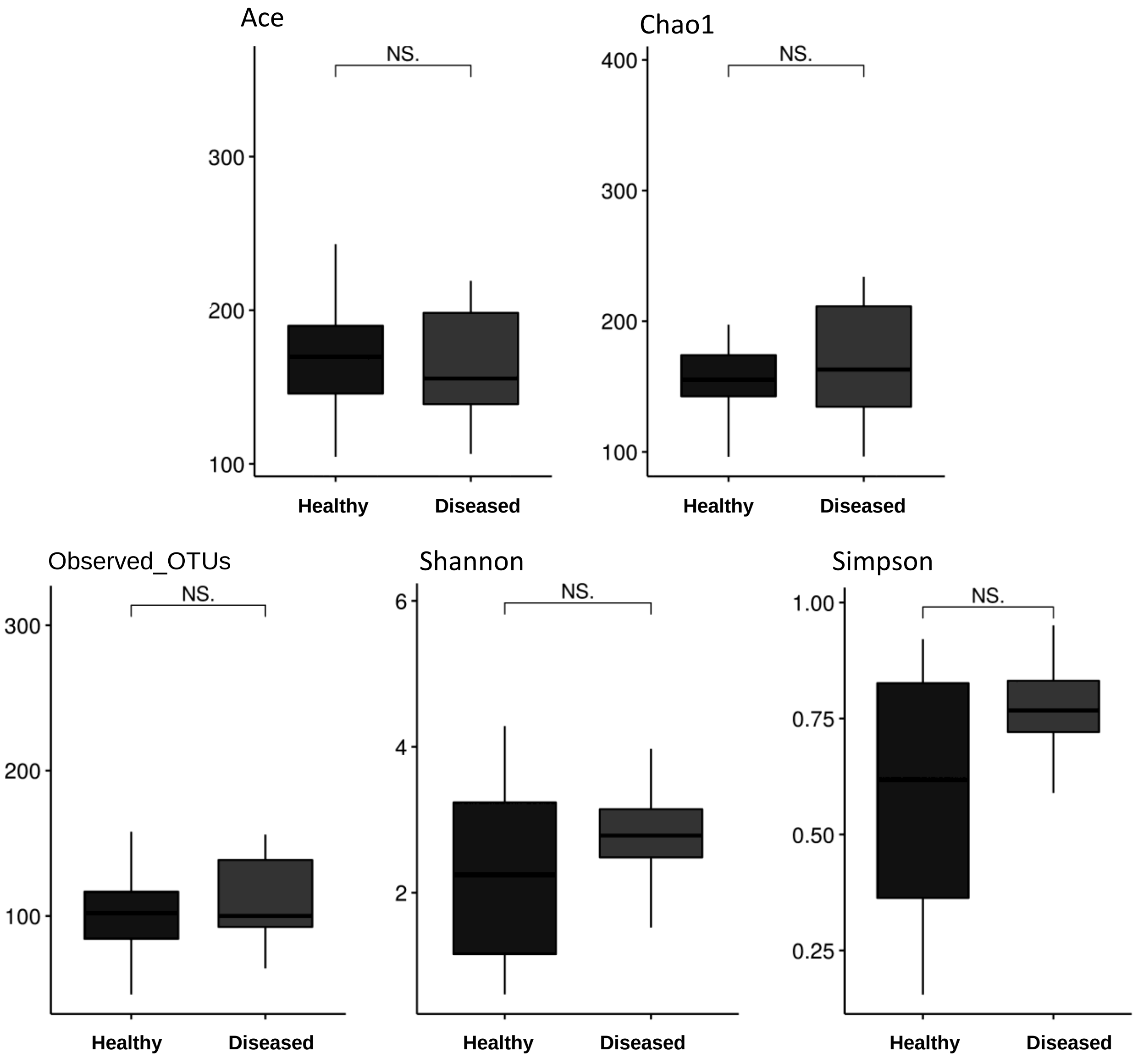

Figure S3

Comparison of alpha diversity indexes at day 7 in the healthy and diseased groups. No significant differences of were observed between two groups.

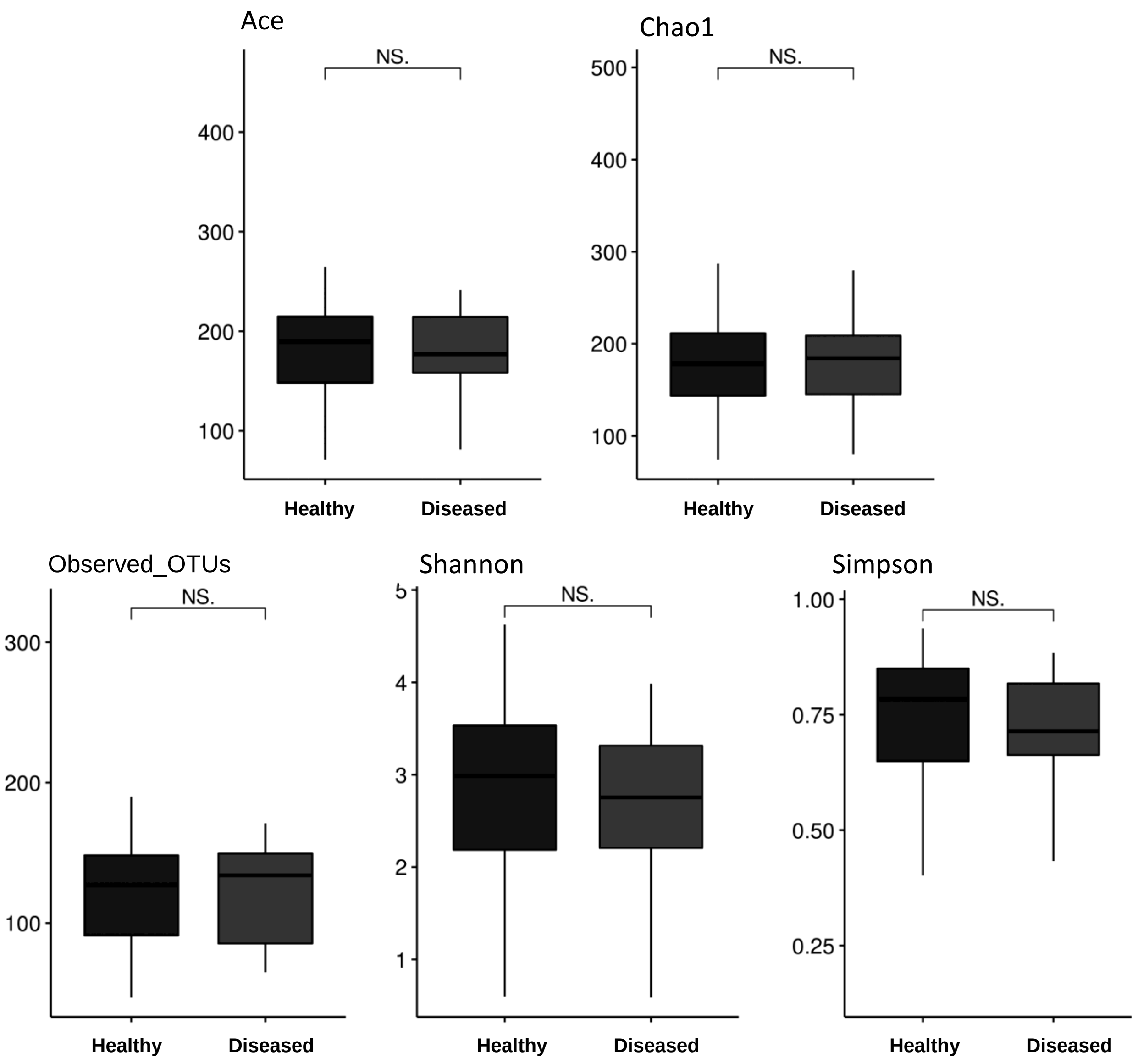

Figure S4

Comparison of alpha diversity indexes at day 15 in the healthy and diseased groups. No significant differences of were observed between two groups.

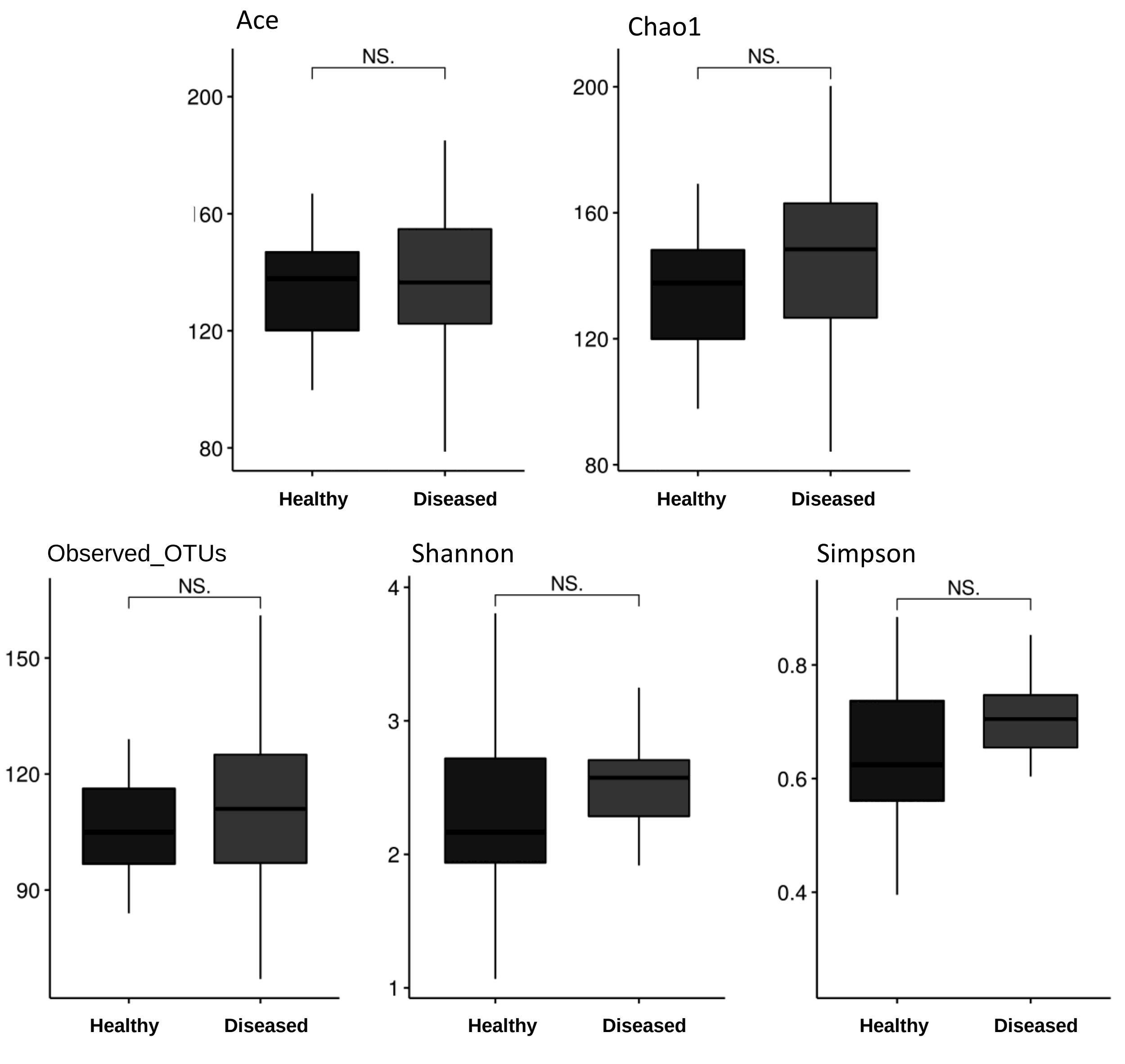

Figure S5

Comparison of alpha diversity indexes at month 1 in the healthy and diseased groups. No significant differences of were observed between two groups.

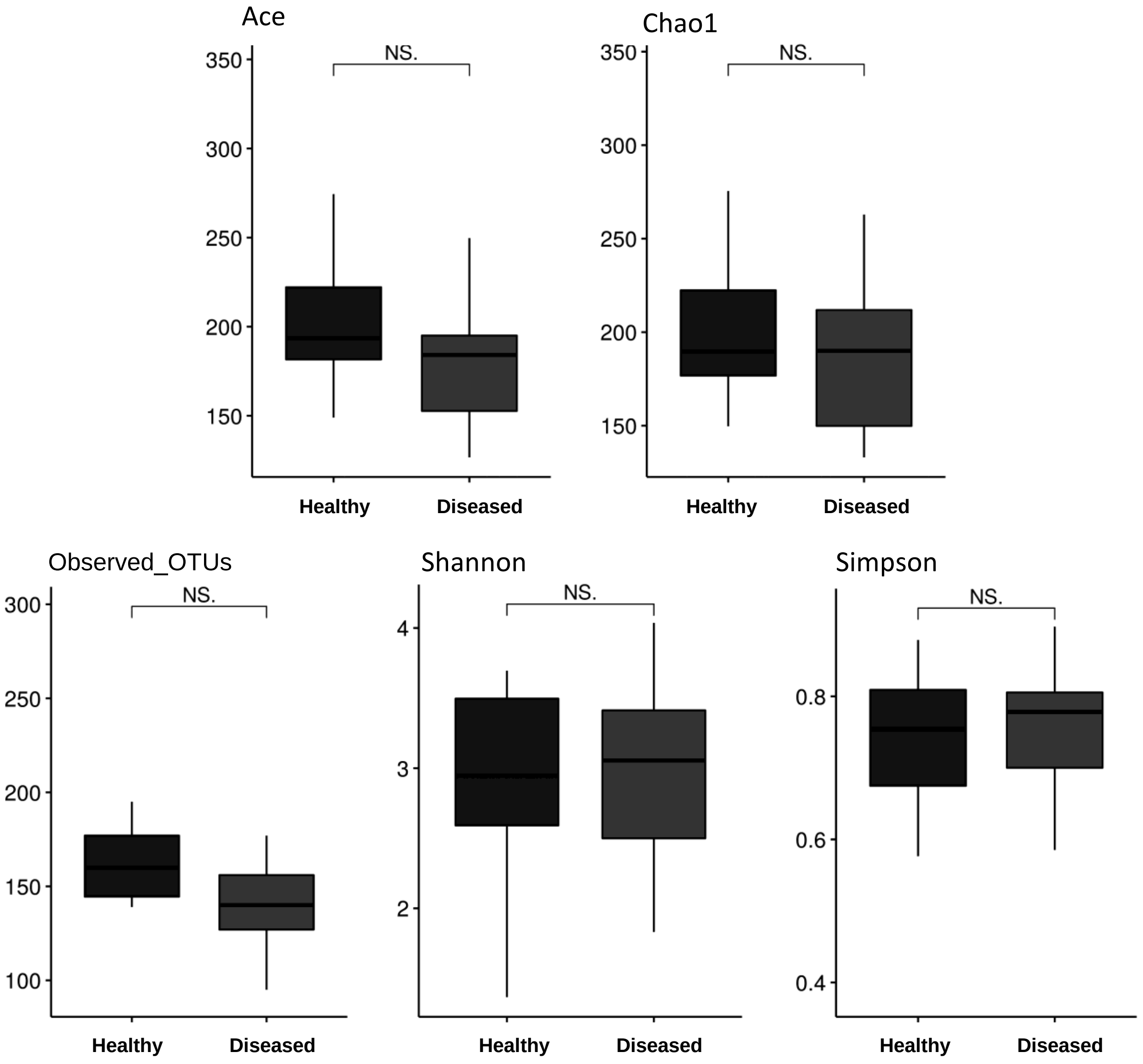

Figure S6

Comparison of alpha diversity indexes at month 6 in the healthy and diseased groups. No significant differences of were observed between two groups.

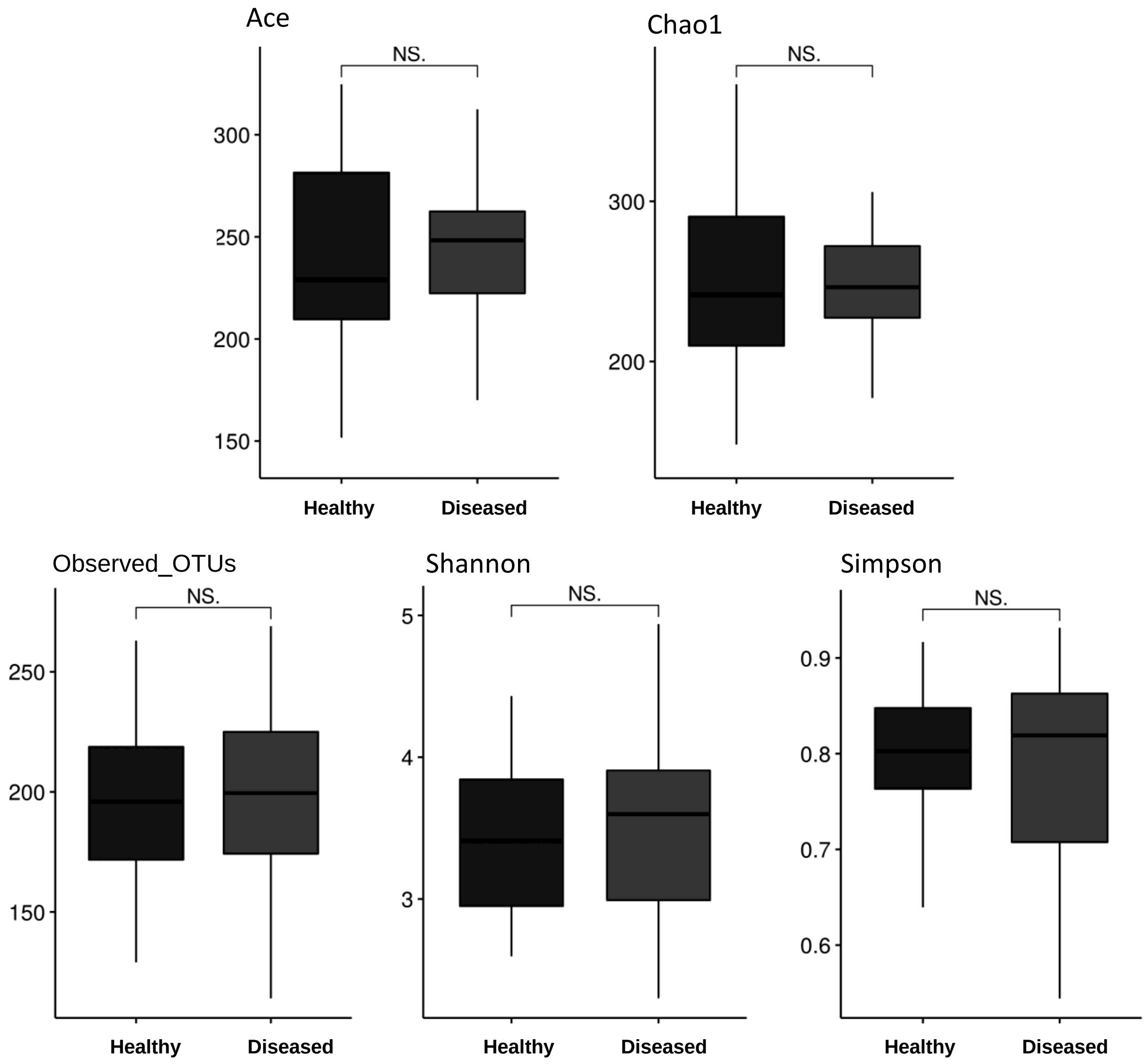

Figure S7

Comparison of alpha diversity indexes at month 12 in the healthy and diseased groups. No significant differences of were observed in two groups.

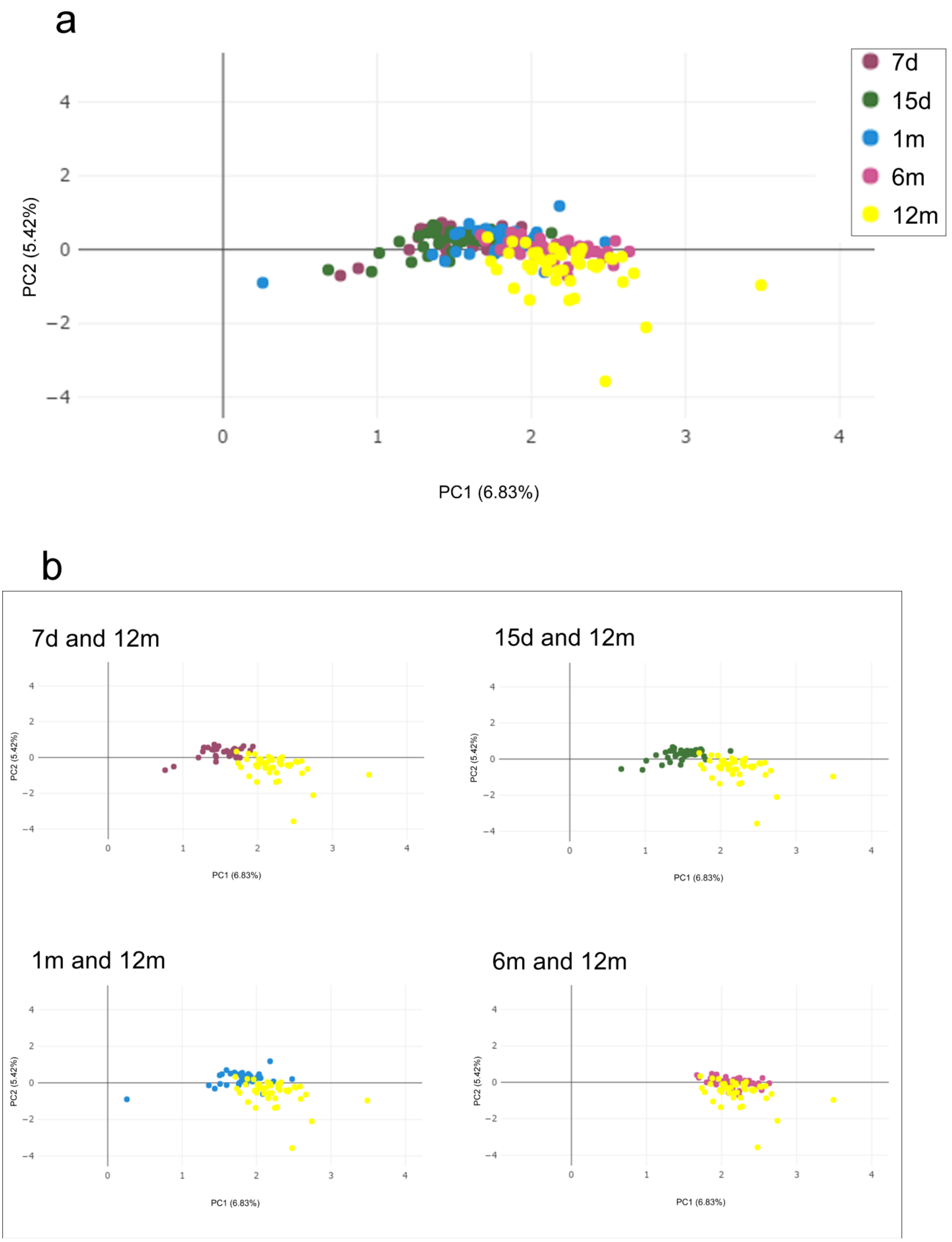

Figure S8

Beta diversity of day 7 to month 12. PLS-DA was carried out according to time group.

a Samples from day 7 to month 12 could not be clearly divided by PLS-DA.

b Trend of beta diversity variation from day 7 to month 12.

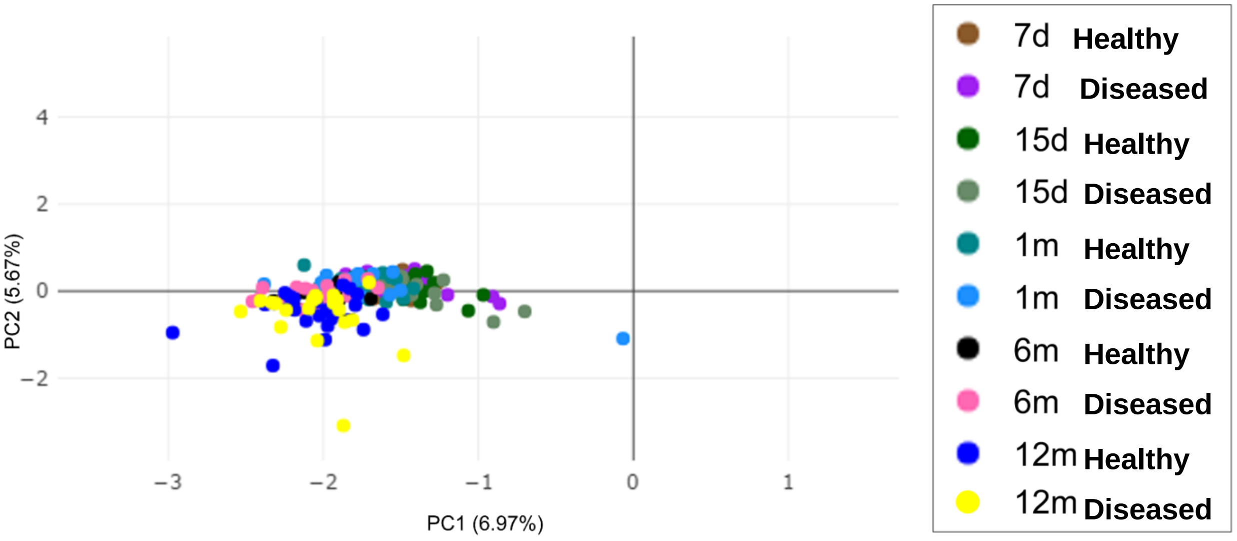

Figure S9

Beta diversity of the healthy and diseased groups. PLS-DA was carried out according to time and diseased groups, and the results reflect beta diversity which is about differences in community structure between these groups. In day 7 to month 12, samples of the healthy and diseased groups could not be distinguished.
